# Supplementary material for: Vaccination coverage against COVID-19 among rural population in Haryana, India: A cross-sectional study
Source: PLoS One. 2024 Mar 8;19(3):e0299564. doi: 10.1371/journal.pone.0299564 (PMC10923481; doi:10.1371/journal.pone.0299564)
Supplement: Attachment — Submitted filename: Rebuttal letter_17_01.docx [file pone.0299564.s003.docx]

**Responses to Reviewers**

**To	Date: 17-01-24**

**The Editor-in-Chief**

**PLOS ONE**

Dear Editor

Thank you and all reviewers for their invaluable suggestions which help us to improve the manuscript to a great extent.

We have submitted the revised manuscript as suggested by the reviewers’ comments.

Looking forward to your response on our resubmission

Kind regards

Dr. Suraj Singh Senjam [corresponding author]

Contact: Suraj Singh Senjam, ORCID ID: <https://orcid.org/0000-0001-8204-3669>

Community Ophthalmology, Dr. Rajendra Prasad Centre for Ophthalmic Sciences,

All India Institute of Medical Sciences, New Delhi, India

Tele [O]: +91-011-2659-3032

Mobile: +91 9968856431

Email ID: [drsurajaiims@gmail.com](mailto:drsurajaiims@gmail.com)

Responses to Reviewers

The authors would like to thank the editor and all reviewers for their time spent reading the manuscript and for helping us to improve the manuscript further. We have revised it as per advice. The revisions are highlighted.

**Comments**

Reviewer #5: Dear Authors, Good that many suggested changes are incorporated and revised.

page 6 Study tool section: More importance is given to the data management platform- Survey Monkey but the study tool is the questionnaire used in the study. This will create ambiguity for the readers.
Page 7- Pilot testing is mentioned but the number or percentage of pilot tested population is missing.

**Reply**

Thank you, reviewer. You are right. SurveyMonkey is not a survey tool, rather it is a software (cloud-based) that can be used to develop survey questionnaires. Correction has been done on page 7. During the pilot testing, we covered 10 Households ( 31 eligible participants)

**Comments**

Reviewer #13: Dear Author,
All the comments and suggestions are well addressed and the manuscript is written well. However, you should also discuss more about the factors like set up of vaccination program, factors, coordination, people awareness, etc which may explain this large vaccination coverage for COVID-19.

**Reply**

We are grateful to you for your advice and suggestions. We have incorporated the suggested points on page 26. We also added about CoWIN app since it is easy to use and user friendly.
